# Alpha-PET with terbium-149: evidence and perspectives for radiotheragnostics

**DOI:** 10.1186/s41181-016-0008-2

**Published:** 2016-03-28

**Authors:** Cristina Müller, Christiaan Vermeulen, Ulli Köster, Karl Johnston, Andreas Türler, Roger Schibli, Nicholas P. van der Meulen

**Affiliations:** 1 Center for Radiopharmaceutical Sciences ETH-PSI-USZ, Paul Scherrer Institut, Villigen-PSI, Switzerland; 2 Institut Laue-Langevin, Grenoble, France; 3 Physics Department, ISOLDE/CERN, Geneva, Switzerland; 4 Laboratory of Radiochemistry, Paul Scherrer Institut, Villigen-PSI, Switzerland; 5 Department of Chemistry and Biochemistry, University of Bern, Bern, Switzerland; 6 Department of Chemistry and Applied Biosciences, ETH Zurich, Zurich, Switzerland

**Keywords:** ^149^Tb, radiolanthanide, mass separation, PET imaging, α-radionuclide therapy, DOTANOC, AR42J tumor

## Abstract

^149^Tb represents a powerful alternative to currently used α-emitters: the relatively short half-life (T_1/2_ = 4.1 h), low α-energy (3.97 MeV, I_α_ = 16.7 %), absence of α-emitting daughters and stable coordination via DOTA are favorable features for potential clinical application. In this letter, we wish to highlight the unique characteristics of ^149^Tb for PET imaging, based on its positron emission (E_β+mean_ = 730 keV, I_β+_ = 7.1 %) in addition to it’s a therapeutic value. To this end, a preclinical study with a tumor-bearing mouse is presented. The perspective of alpha-PET makes ^149^Tb highly appealing for radiotheragnostic applications in future clinical trials.

## Correspondence/Findings

Dear Editor

Recently, α-radionuclide therapy has gained in popularity and attracted the interest of physicians [1, 2]. One of the reasons is certainly the success of Xofigo^TM^ (^223^RaCl_2_), which has been approved for the treatment of patients with symptomatic bone lesions in castration-resistant prostate cancer [3]. The survival benefit after Xofigo^TM^ therapy, combined with its low toxicity, make it undoubtedly very promising as a novel treatment option for this type of disease and, in future, also possibly for patients suffering from bone metastases of other cancer types [3]. Also, the power of targeted therapy with other α-particle emitters, namely, ^211^At, ^225^Ac and ^213^Bi has been convincingly demonstrated, mostly in combination with tumor-targeted antibodies [4].

α-Particles are positively-charged and have a shorter tissue range (~25–80 μm) and much higher energies (~4-8 MeV), as compared to the negatively-charged β^-^-particles of clinically useful β^-^-emitters [1]. In terms of radiobiological effects, it is important to mention that the linear energy transfer (LET) of α-particles is with ~100 keV/μm very high and may further increase up to 300 keV/μm towards the end of the track (Bragg peak) [5]. This value is far above the LET (~0.2 keV/μm) of β^-^-particles. High-LET radiation generally causes more lethal cell damage than low-LET radiation due to the formation of more irreparable double-strand DNA breaks [6]. All these characteristics make targeted α-therapy best suited for specific tumor cell killing, without collateral damage of surrounding healthy tissue [1, 7]. Single photon emission computed tomography (SPECT) may be used to image targeted α-particle therapy if the decay of the α-emitting radionuclide results in photon emission of a suitable energy and sufficient intensity [4]. This is the case for ^223^Ra (E_γ_ = 144 keV, 3.3 %; 154 keV, 5.7 %) [8], while ^225^Ac may potentially be imaged via γ-ray emission of daughter radionuclides (e.g. ^221^Fr, E_γ_ = 218 keV, 11 %), as shown in preclinical settings [9], and ^211^At through use of emitted X-rays [10]. The low quantities of activity employed for α-therapy using these radionuclides, remain, however, challenging for nuclear imaging using SPECT.

^149^Tb represents a powerful alternative to the currently-employed α-emitters. It decays with a relatively short half-life of 4.1 h and emits α-particles of low energy (E_α_ = 3.97 MeV, I_α_ = 16.7 %), resulting in a tissue range of ~25 μm and a LET of 140 keV/μm [11]. These physical properties make it particularly well suited for application in combination with small-molecular-weight targeting agents, including peptides, which are quickly cleared from the body [12]. The absence of α-emitting daughters is regarded as an additional favorable feature of ^149^Tb, since toxicity of α-emitters with multiple α-emitting daughters has been identified as an issue for clinical application [2]. In vivo application of ^149^Tb may, thus, be feasible without the risk of unspecific emission of harmful α-particles in the body as a consequence of released daughter radionuclides. The decay scheme of ^149^Tb is, nevertheless, complex [7] and potential radiotoxicity of the resulting radiolanthanides remains to be determined. As a radiolanthanide ^149^Tb can be stably coordinated with the conventional macrocyclic 1,4,7,10-tetraazacyclododecane-1,4,7,10-tetraacetic acid (DOTA) chelator [13, 14]. These circumstances allow the use of ^149^Tb with DOTA-functionalized compounds that are (pre)clinically established for ^177^Lu-based radionuclide therapy.

Beyer et al. reported on a preclinical immunotherapy with ^149^Tb-labeled rituximab (5.5 MBq/mouse) in a mouse model of Daudi cell-based lymphoma [15]. In this case, the open-chained cyclohexane diethylene triamine pentaacetic acid (CHX-A”-DTPA) chelator was used for radiometal coordination. The majority (89 %) of treated mice showed tumor-free survival over >120 days, while all untreated controls and mice which received unlabeled rituximab developed lymphoma disease [15]. Our group has previously reported on a preclinical pilot study using a ^149^Tb-labeled folate conjugate for therapy of mice bearing folate receptor-positive KB tumor xenografts [14]. In treated mice (2.2 MBq and 3.0 MBq/mouse, respectively), the tumor growth was significantly delayed which prolonged the average survival time to 30.5 days and 43 days, respectively, compared to untreated controls which survived only 21 days on average [14].

Even though the number of preclinical studies in which ^149^Tb was investigated, is very small, there is clear evidence of the potential to use ^149^Tb for targeted α-radionuclide therapy. Other than the aforementioned favorable physical and chemical characteristics it provides, it would also offer a unique opportunity for comparing the effects of α-radionuclide therapy with β^-^-radionuclide therapy through the use of chemically identical radiopharmaceuticals labeled with ^149^Tb and ^161^Tb (T_1/2_ = 6.9 d, E_β-mean_ = 154 keV) [13]. As all of these features of ^149^Tb are so attractive from a therapy perspective, the proposed possibility of positron emission tomography (PET) provides an extra dimension. Alternatively, ^149^Tb may also be used for SPECT imaging based on the emission of γ-radiation of a suitable energy and reasonable intensity (E_γ_ = 165 keV, I_γ_ = 26.4 %). This concept has been proposed earlier [16], but has to date not been investigated in preclinical studies. The current trend in nuclear medicine is, however, in favor of PET instead of SPECT due to the higher sensitivity and resolution it provides [17].

In a recently-performed study, we focused on the potentially unique characteristic of ^149^Tb to be used for PET imaging, based on its positron emission (E_β+mean_ = 730 keV, I_β+_ = 7.1 %), in addition to its α-therapeutic value [16]. ^149^Tb was produced by proton-induced spallation of a tantalum target, followed by an online isotope separation process at ISOLDE/CERN (Geneva, Switzerland). The mass-separated ion beam was implanted into a zinc-coated gold catcher foil, which was shipped to Paul Scherrer Institut (PSI, Villigen-PSI, Switzerland) for processing. ^149^Tb was separated from isobar and pseudo-isobar impurities by cation exchange chromatography, as previously reported [13]. The separation yield was 100 MBq (~99 %) of highly pure ^149^Tb in α-hydroxyisobutyric acid solution (pH 4.7), a quantity sufficient for preclinical application. The radiolabeling was carried out directly in the eluent solution by addition of DOTANOC and incubation of the reaction mixture for 15 min at 95 °C. ^149^Tb-DOTANOC was obtained with >98 % radiochemical purity at a high specific activity (5 MBq/nmol), as confirmed by high performance liquid chromatography (HPLC)-based quality control. A nude mouse bearing AR42J tumor xenografts was intravenously injected with ~7 MBq ^149^Tb-DOTANOC (~1.4 nmol). PET/CT scans were performed 2 h later using a preclinical G8 bench-top scanner (Sofie Biosciences). During the PET scan (30 min) and the following CT (1.5 min) the mouse was anesthesized using a mixture of isoflurane and oxygen.

**Fig. 1:**
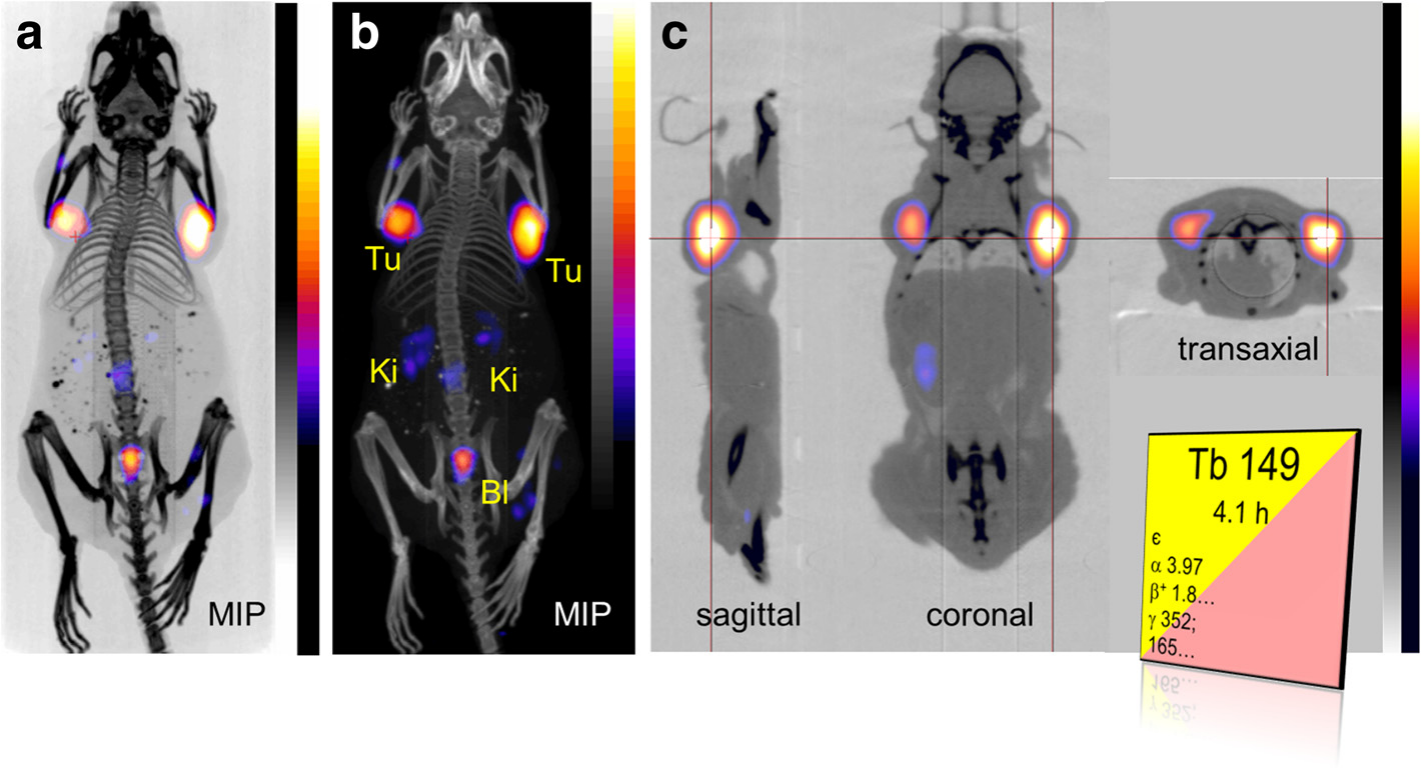
PET/CT images of an AR42J tumor-bearing mouse 2 h after injection of ^149^Tb-DOTANOC (7 MBq). (**a**, **b**) Maximal intensity projections (MIP) and (**c**) sections showed distinct accumulation of radioactivity in tumor xenografts (Tu) and residual radioactivity in kidneys (Ki) and urinary bladder (Bl). The decay scheme of ^149^Tb is shown as in the Karlsruhe Nuclide Chart (www.nucleonica.com)

The quality of the obtained PET images was unexpectedly high (Fig. 1). The maximal intensity projections allowed distinct visualization of the tumors located on each shoulder (Fig. 1a/b). Specific cross sections of the tumor showed homogenous distribution of radioactivity accumulation (Fig. 1c). Residual radioactivity was found in the kidneys and the urinary bladder as expected, based on the fast renal excretion of DOTANOC.

In this study, the possibility of being able to produce a PET image using a ^149^Tb-labeled biomolecule was successfully demonstrated. It is, thus, indisputable that ^149^Tb presents an exceptional potential to be used in clinics as it would allow combining α-therapy with PET using a single radionuclide.

It has to be acknowledged, however, that the quantity of injected activity for patients may be critical for PET imaging purposes. So far, it is unknown how much activity would be required for a therapeutic application in the clinics. Among several parameters, including the sensitivity of tumor type which should be treated, it will be critically dependent on the targeting agent and the degree of its accumulation in the tumor tissue. Whether the necessary quantity of radioactivity would allow for PET imaging remains to be determined in patients.

The unconventional production of ^149^Tb appeared to be the main reason why ^149^Tb did not yet reach clinical trials, as stated in several reports previously [12]. Currently, endeavors all over the world are focused on the establishment of new radionuclide production centers, clearly offering new perspectives for the production of radionuclides like ^149^Tb, which are dependent on mass separation facilities. Such production centers, which exploit spallation production combined with isotope separation on-line (ISOL), are already in operation at the *Isotope Separator and Accelerator* (ISAC) at TRIUMF, Canada’s National Laboratory for Particle and Nuclear Physics (Vancouver, Canada) and at *Investigation of Radioactive Isotopes on Synchrocyclotron* (IRIS), at the Petersburg Nuclear Physics Institute (PNPI, Gatchina, Russia). Other facilities are in the planning stage or under construction at *Radioactive Isotope Beam Factory* (RIBF, East Lansing, U.S.), at the Belgium Nuclear Research Center’s ISOL facility (*ISOL@MYRRHA*, Mol, Belgium) and at the *Japan Proton Accelerator Research Complex* (J-PARC ISOL, Tokai, Japan). MEDICIS, a new radionuclide production center dedicated to medical applications, is currently being built at CERN (Geneva, Switzerland) [12]. MEDICIS’ aim is to produce medically interesting, but not yet fully investigated radionuclides, including ^149^Tb, in quantities sufficient to address the requirements of pilot investigations in patients.

The perspective of overcoming the obstacle of production holds great promise for more detailed preclinical investigations and first clinical trials in the near future using ^149^Tb for α-therapy, combined with PET.
